# The failing human heart is characterized by decreased numbers of telocytes as result of apoptosis and altered extracellular matrix composition

**DOI:** 10.1111/jcmm.12664

**Published:** 2015-08-26

**Authors:** Manfred Richter, Sawa Kostin

**Affiliations:** a Department of Cardiac Surgery, Kerckhoff-ClinicBad Nauheim, Germany; b Max-Planck-Institute for Heart and Lung ResearchBad Nauheim, Germany

**Keywords:** telocytes, telopodes, extracellular matrix, myocardial remodelling, heart failure

## Abstract

Telocytes (TCs) are a novel type of interstitial cells only recently described. This study aimed at characterizing and quantifying TCs and telopodes (Tps) in normal and diseased hearts. We have been suggested that TCs are influenced by the extracellular matrix (ECM) composition. We used transmission electron microscopy and c-kit immunolabelling to identify and quantify TCs in explanted human hearts with heart failure (HF) because of dilated, ischemic or inflammatory cardiomyopathy. LV myectomy samples from patients with aortic stenosis with preserved ejection fraction and samples from donor hearts which could not be used for transplantation served as controls. Quantitative immunoconfocal analysis revealed that 1 mm^2^ of the normal myocardium contains 14.9 ± 3.4 TCs and 41.6 ± 5.9 Tps. As compared with the control group, the number of TCs and Tps in HF decreased more than twofold. There were no differences between HF and control in the number of Ki67-positive TCs. In contrast, terminal deoxynucleotidyl transferase-mediated dUTP nick end labelling-positive TCs increased threefold in diseased hearts as compared to control. Significant inverse correlations were found between the amount of mature fibrillar collagen type I and the number of TCs (*r* = −0.84; *P* < 0.01) and Tps (*r* = −0.85; *P* < 0.01). The levels of degraded collagens showed a significant positive relationship with the TCs numbers. It is concluded that in HF the number of TCs are decreased because of higher rates of TCs apoptosis. Moreover, our results indicate that a close relationship exists between TCs and the ECM protein composition such that the number of TCs and Tps correlates negatively with the amount of mature fibrillar collagens and correlates positively with degraded collagens.

## Introduction

Telocytes (TCs) are a novel type of interstitial cells 1–17. The ultrastructure using transmission electron microscopy (TEM) of TCs is now very well studied. Telocytes in different organs share similar features 7–18 and are characterized by having very small cell bodies (consisting of a nucleus and a small amount of cytoplasm and extremely long (up to 100 μm) and thin processes called telopodes (Tps). Telopodes consist of long thin tubes (called podomers) interspersed with dilations (called podoms) which contain mitochondria and caveolae. Calcium-release units including caveolae, sarcoplasmic reticulum and mitochondria are also typical for TCs. Another peculiar feature of TCs is the presence of labyrinthine systems interconnecting Tps from the same TC or from neighbouring TCs 4,19. Telocytes have been identified in numerous organs including the heart (for review see Ref. 1,20–22).

In cardiac pathologies, TCs have been described in atrial amyloidosis in human patients 23, systemic sclerosis 24 and in experimental myocardial infarction 25–27. Nevertheless, more studies are needed to investigate the role of TCs in different cardiac diseases leading to heart failure (HF) in human patients. Therefore, the purpose of this study was to investigate by TEM and immunoconfocal microscopy, the structure and number of TCs in human hearts fail as a result of different forms of cardiomyopathies. In addition, a recent *in vitro* study using isolated TCs has shown that the dynamics of cardiac Tps can be influenced *in vitro* by the protein composition of the extracellular matrix (ECM) 28. Therefore, we have been suggested that the composition of the ECM might also influence the distribution pattern and the number of TCs in diseased human hearts. Specifically, we embarked on correlating the number of TCs and Tps with mature fibrillar collagens and with degraded collagen type I and type III.

## Material and methods

### Patients

LV samples were collected from the explanted hearts of patients who underwent orthotopic transplantation because of end-stage HF. All patients were severely symptomatic [New York Heart Association (NYHA) grade IV]. Patients either had normal coronary arteries with histologically proven chronic myocarditis (MYO, *n* = 6) according to the criteria of the Society for Cardiovascular Pathology 29, or were diagnosed as idiopathic dilated cardiomyopathy (DCM, *n* = 6), or ischemic cardiomyopathy (ICM, *n* = 7). Diagnosis of ICM was based on clinical history, Doppler echocardiography and cardiac catheterization data. Patients were functionally classified according to criteria of the NYHA and were listed for heart transplantation according to the guidelines for organ transplantation of the German Medical Association. Clinical data are summarized in Table1. All patients before transplantation had a standard medication regimen including diuretics, digitalis, ACE inhibitors and β-blockers. Control tissues consisted of: (*i*) LV samples from 4 patients subjected to subvalvular myectomy during surgical aortic valve replacement, and (*ii*) LV samples from 2 donor hearts with normal LV function which could not be used for transplantation. Patients with aortic valve stenosis showed preserved cardiac function (ejection fraction ≥60%), and the resected myocardial tissue did not present signs of ischemic damage or myocardial hypertrophy. The institutional Ethical Committee approved the study, and all patients have given written informed consent.

**Table 1:** Clinical data

	Control	DCM	ICM	MYO
Number	6	6	7	6
Age (years)	50.4 ± 5.8	49.3 ± 6.7	54.8 ± 2.4	43.4 ± 5.8^*^
Sex (male/female)	5/1	6/0	7/0	4/2
NYHA class, *n*	–	IV (6/6)	IV (7/7)	IV (6/6)
EF (%)	>60%	15 ± 2.1	21 ± 2.2	23 ± 4.3
Medications				
ACE inhibitors (*n*)	2	3	3	3
Beta-blockers (*n*)	4	2	2	2
Ca^2+^ blockers (*n*)	2	2	2	2
Digitalis (*n*)	1	5	5	4
Diuretics (*n*)	1	5	6	5

ACE: agiotensin converting enzyme; EF: ejection fraction, NYHA: New York Heart Association; ICM: ischemic cardiomyopathy; DCM: dilated cardiomyopathy; MYO: myocarditis. ^*^P < 0.05 versus ICM.

### Tissue sampling

LV specimens were dissected into smaller pieces and fixed in 2% glutaraldehyde for TEM or immediately frozen in liquid nitrogen and stored at −80°C. From patients with ICM we have investigated the myocardium distant from the post-infarction scar tissue.

### Transmission electron microscopy

Specimens pre-fixed in 2% glutaraldehyde were post-fixed in 2% osmium tetroxide for 1 hr. After dehydration in graded concentrations of alcohol and propylene oxide, they were embedded in Epon, following routine procedures. Semithin (1 μm) sections were stained with toluidine blue and viewed in a Leica DM microscope (Leica Microsystems, Wetzlar, Germany). Ultrathin sections were stained with uranyl acetate and lead citrate, and viewed and photographically recorded under a Philips CM10 electron microscope (Eindhoven, the Netherlands).

### Immunofluorescent labelling and confocal microscopy

The tissue samples were mounted in Tissue-Tek^®^ O.C.T.^™^ compound (Sakura Finetek, Tokyo, Japan) and cryosections 5 μm thick were prepared. Cryosections were air-dried and fixed for 10 min. in either acetone (−20°C), 4% paraformaldehyde (room temperature) or Carnoys' solution (room temperature). After washing in PBS sections were incubated with 1% bovine serum albumin for 30 min. to block non-specific binding sites. After rinsing in PBS, the samples were incubated overnight with primary antibodies against c-kit (catalogue number A4502 and clone 104D2; Dako, Clostrup, Denmark), Ki67 (clone MIB-1; Dako) and collagen I (Clone COL-1; Sigma-Aldrich, St Louis, MO, USA). Antibodies against carboxyterminal telopeptide (ICTP), N-terminal propeptide I (PINP) and N-terminal propeptide III (PIIINP) were prepared and used as previously described 30–33. Antimouse or anti-rabbit IgG-conjugated with Cy3 or Cy2 (Jackson Immunoresearch Laboratories, West Grove, PA, USA) served as detection systems in single or double immunolabellings. The nuclei were stained with 1 μg/ml 4,6-diamidino-2-phenylindole (DAPI; Molecular Probes, Leiden, the Netherlands). F-actin was fluorescently stained using FITC- (Sigma-Aldrich) and Alexa633-conjugated phalloidin (Molecular Probes). Negative controls were obtained by omitting the primary antibody, in an otherwise similar protocol. The terminal deoxynucleotidyl transferase-mediated dUTP nick end labelling (TUNEL) reaction was performed as previously described 34. The samples were examined by confocal laser microscopy using Leica TCS2 and Leica TCS8 microscopes (Leica Microsystems). Digital images were further processed for 3D-reconstruction using Imaris^®^ 7.4.2 software (Bitplane AG, Zürich, Switzerland).

### Quantitative immunofluorescent microscopy

Measurements of immunofluorescence for collagen I and markers of collagen metabolism were carried out using a ×40 Planapo objective and a Leica (Leitz DMRB) fluorescent microscope (Leica Microsystems). Cryosections from at least two different tissue blocks in each case were used. For quantitative analysis, all sections were immunolabelled simultaneously using identical dilutions of primary and secondary antibodies and other reagents. Immunofluorescent images were obtained under identical parameters of imaging, zoom, objectives and fluorescence power. Sections exposed to PBS instead of primary antibodies served as negative controls. Image acquisition settings were standardized for all groups to ensure that the image collected demonstrated a full range of fluorescence intensity from 0 to 255 pixel intensity level and were kept constant during all measurements. For each patient, at least 10 random fields of vision were analysed for the quantity of the collagen markers investigated using analysis software (Leica Microsystems) and NIH ImageJ program (http://rsb.info.nih.gov/ij/). The area occupied by different collagen markers was calculated as percentage of collagen I or of collagen markers labelling per tissue area.

To quantify the number of TCs, we used the positive expression of c-kit of cells having a DAPI-positive nucleus and thin prolongations. C-kit-positive prolongations without a DAPI-positive nucleus were considered Tps. Cryosections from at least two different tissue blocks and from ten random fields in each case were used and the number of TCs and Tps were calculated per 1 mm^2^ myocardial area. The number of proliferating and apoptotic TCs was expressed as absolute numbers of, respectively, Ki67- and TUNEL-positive cells per 1000 TCs.

### Statistical analysis

All data are presented as means ± S.D. For multiple comparisons we used anova, followed by analysis with the Bonferroni *t*-test. Associations between variables were assessed using Spearman's rank correlation. Differences between groups were considered significant at *P* < 0.05.

## Results

### Diseased myocardium is characterized by a decreased number of TCs and Tps

**Figure 1:**
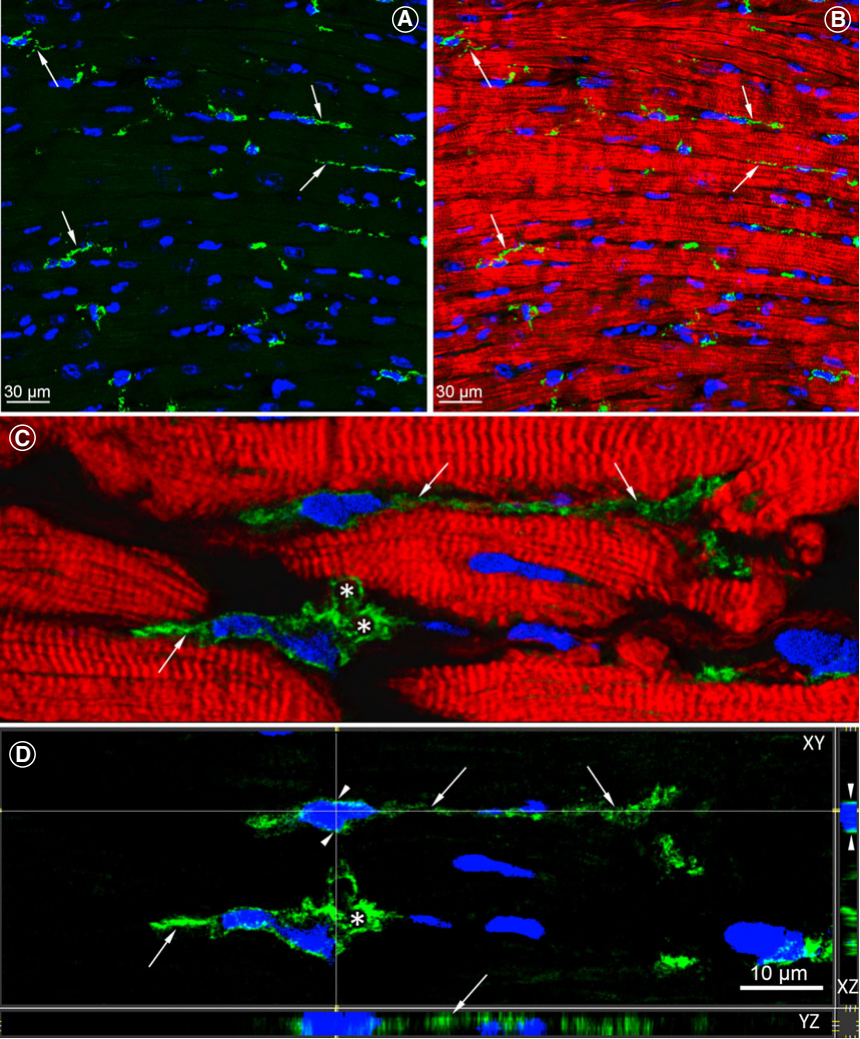
Confocal images of c-kit-positive telocytes (TCs) in longitudinal sections of the normal human LV myocardium. (**A**) Numerous c-kit-positive cells (green) with long cytoplasmic processes are indicated by arrows. (**B**) Merged view of the image shown in **A** showing the spatial relationship of TCs with the neighbouring cardiomyocytes stained red with Alexa633-conjugated phalloidin. (**C**) Two TCs located in close vicinity with cardiomyocytes. Arrows denote telopodes (Tps) originating from the cell body. Asterisks indicate labyrinthine systems. (**D**) Identical images as in **B** viewed in XY, XZ and YZ axes. Arrowhead points the very small rim of the perinuclear cytoplasm. Thin and long TPs are indicated with arrows and the asterisk indicates the labyrinthine system. Nuclei are stained blue with 4,6-diamidino-2-phenylindole (DAPI).

Immunolabelling for c-kit and confocal microscopy of the control human hearts revealed that the interstitial c-kit-positive TCs with long Tps are in close vicinity or intermingling with cardiomyocytes and form a network-like distribution pattern (Fig.1).

**Figure 2:**
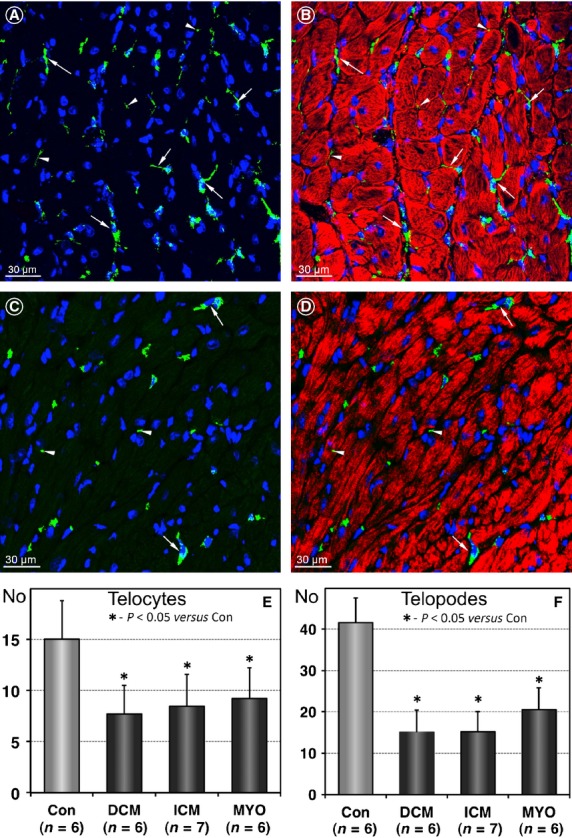
Telocytes (TCs) and telopodes (Tps) are reduced in diseased human hearts. (**A** and **B**) Representative immunofluorescent confocal images showing the distribution of c-kit-positive cells (green) in a normal heart. Arrows denote TCs and arrowheads indicate TPs. (**C** and **D**) Confocal images of TCs in a patient with DCM demonstrating fewer TCs (arrows) and TPs (arrowheads) than in controls. Cardiomyocytes are stained red with Alexa633-conjugated-phalloidin. Nuclei are stained blue with 4,6-diamidino-2-phenylindole (DAPI). (**E** and **F**) Results of quantification of the number of TCs and TPs per 1 mm^2^ myocardial area in controls and heart failure patients.

Confocal images presented in Figure2 compare the number of TCs in normal control hearts (Fig.2A and B) with that in diseased human hearts (Fig.2C and D). These examples show that the number of TCs is apparently diminished in diseased hearts as compared with controls. Quantitative analysis revealed that 1 mm^2^ of normal myocardium contains 14.9 ± 3.4 TCs and 41.6 ± 5.9 Tps (Fig.2E and D). In HF, the number of TCs decreased significantly and averaged 7.7 ± 0.7 in DCM, 8.4 ± 3.1 in ICM and 9.16 ± 2.9 TCs per 1 mm^2^ myocardial area in patients with MYO (Fig.2E). Similarly, the number of Tps in diseased hearts TCs decreased more than twofold as compared with the control values (Fig.2D).

**Figure 3:**
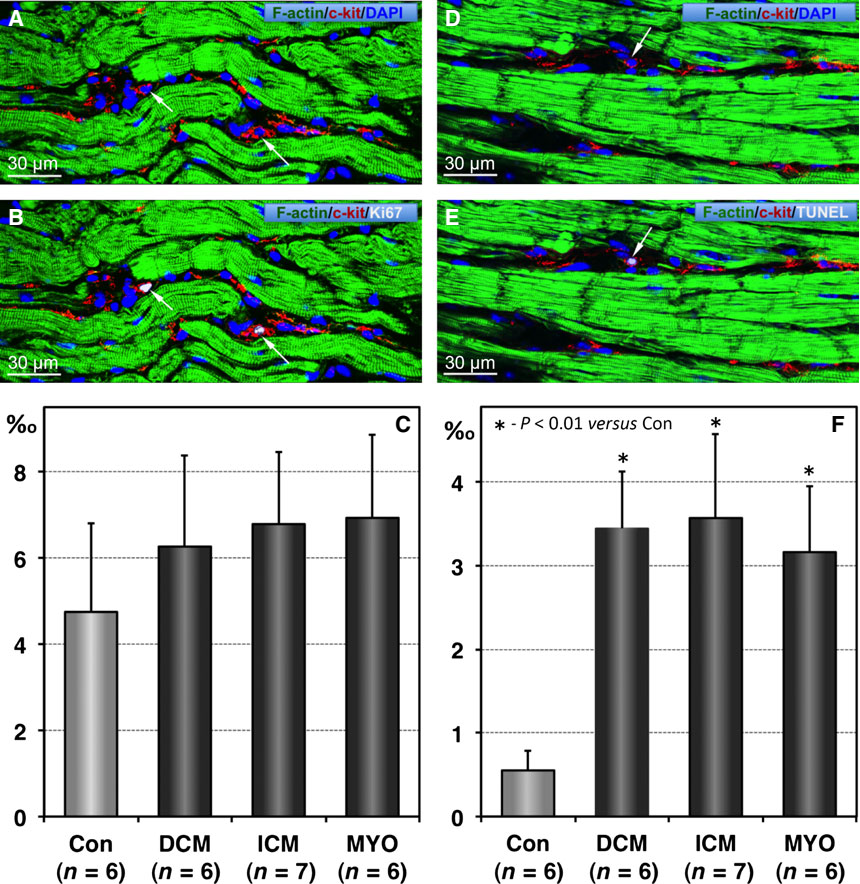
Proliferation and apoptosis of telocytes (TCs). (**A** and **B**) Confocal images showing typical TCs (red) in a patient with chronic myocarditis and heart failure. Arrows indicate 2 TCs that are Ki67-positive. Cardiomyocytes are stained green with phalloidin and nuclei are stained blue with 4,6-diamidino-2-phenylindole (DAPI). (**C**) The mean values of Ki67-positive TCs expressed as absolute numbers of such cells per 1000 TCs. (**D** and **E**) Confocal images showing typical TCs (red) being TUNEL-positive (arrow). Cardiomyocytes are stained green with phalloidin and nuclei are stained blue with DAPI. (**F**) Quantification of TUNEL-positive TCs.

Given that the total number of cells in an organ depends on the balance between cell proliferation and cell death we have performed double labellings for c-kit with either Ki-67 or TUNEL reaction. Figure3A and B show an example of c-kit-positive TCs that are also positive for the proliferation marker Ki67. Quantitative analysis revealed that in control hearts only 4.7 ± 1.9 TCs from 1000 TCs are positive for Ki67 (Fig.3C). The number of Ki67-positive TCs in diseased hearts was increased by 30–45% as compared with controls. The difference was not statistically significant. Figure3D and E show a c-kit-positive TC undergoing apoptosis being positive for the TUNEL reaction. Quantitative analysis revealed that in control hearts TC apoptosis is a very rare event involving on average less than one TC per 1000 TCs (Fig.3F). In comparison with the control values, in diseased hearts TUNEL-positive TCs increased more than threefold averaging 3.4 ± 0.7 in DCM, 3.6 ± 1.0 in ICM and 3.16 ± 0.8 apoptotic TCs per 1000 TCs in patients with MYO. Taken together, Ki67 and TUNEL labelling data indicate an imbalance between TC proliferation and TC apoptosis in terms that the apoptosis/proliferation ratio is markedly increased in diseased hearts as compared with normal human hearts.

### The number of TCs and Tps depends on the ECM composition

**Figure 4:**
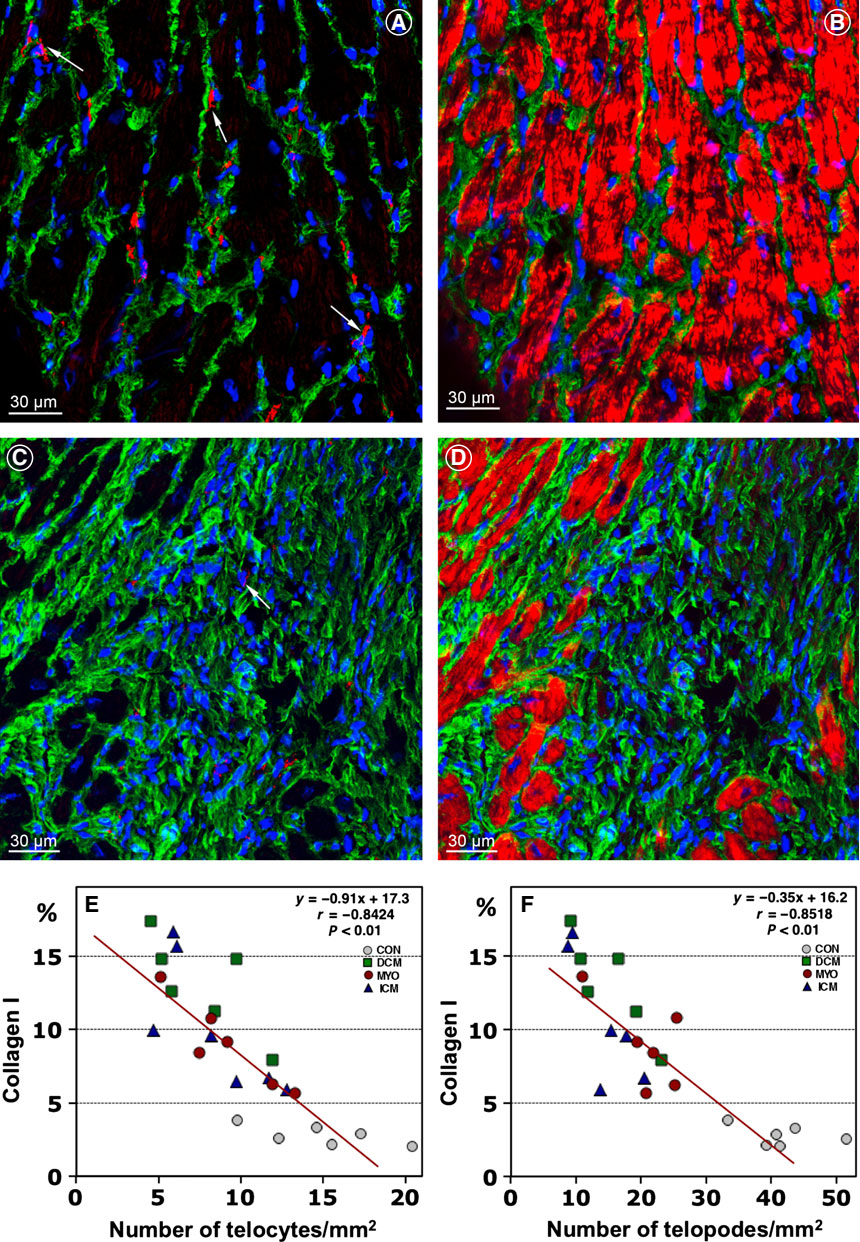
Telocytes (TCs) are decreased in areas of collagen accumulations. Expression of collagen I (green) and the number and distribution of TCs (red) in a control patient (**A** and **B**) and in a patient with ICM (**C** and **D**). Note that TCs and telopodes (Tps) are scarce or absent in the area of replacement fibrosis which contains densely packed collagen fibres (**C** and **D**). Cardiomyocytes are stained red with Alexa633-phalloidin. Nuclei are stained blue with 4,6-diamidino-2-phenylindole (DAPI). (**E** and **F**) Correlations between the percent of collagen I per myocardial area and the number of TCs and Tps.

We have studied the relationship between mature fibrillar collagen type I and TCs using double labelling immunoconfocal microscopy. Images presented in Figure4 compare the amount of collagen I with the number and distribution of TCs in a normal control heart and in a patient with ICM. These examples demonstrate that the interstitial space of the normal human heart contains numerous TCs located in close vicinity to very thin collagen fibrils (Fig.4A and B). In patients with HF, severe collagen deposition in areas of replacement fibrosis is associated with disappearance of the TCs (Fig.4C and D). These observations are supported by the correlations between the percent of myocardial area occupied by collagen I and the number of TCs (Fig.4E) or Tps (Fig.4F). The correlations showed significant inverse relationships between the amount of collagen type I and the number of TCs (*r* = −0.84; *P* < 0.01) and Tps (*r* = −0.85; *P* < 0.01).

**Figure 5:**
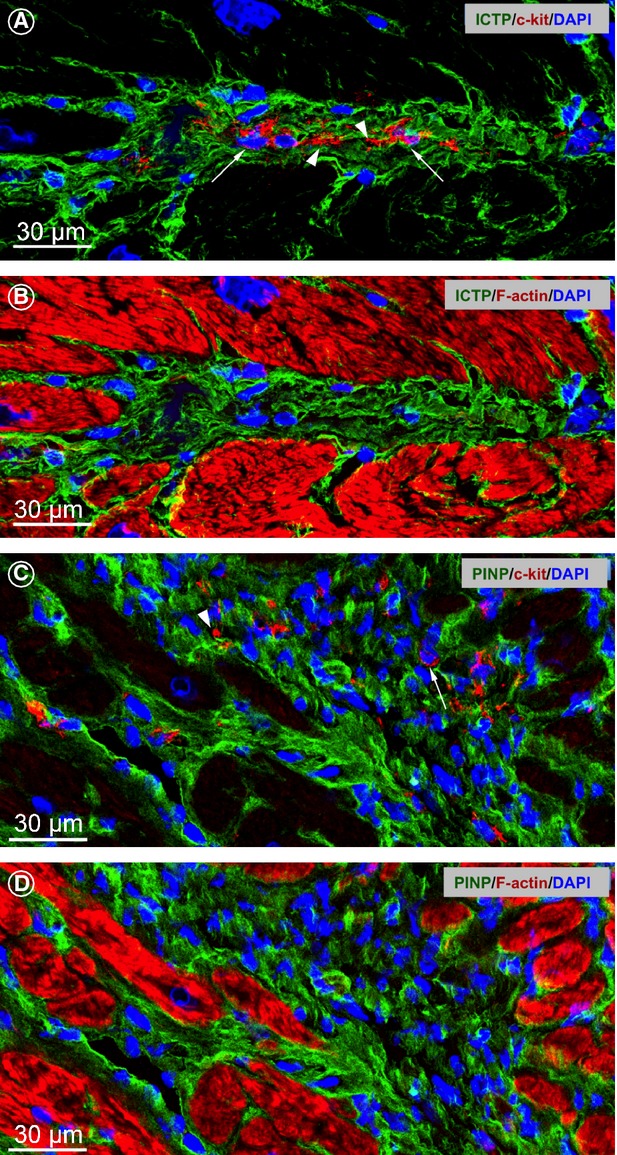
Markers of collagen metabolism and telocytes (TCs). (**A** and **B**) Representative immunofluorescent confocal images showing that accumulation of ICTP (green) is associated with clearly distinguishable TCs (arrows) with long telopodes (Tps; arrowheads) in a patient with chronic myocarditis. (**C** and **D**) Representative immunofluorescent images of a PINP area (green) showing remnants of Tps (arrowhead) in a patient with DCM. Arrow indicates c-kit staining of a TC cell body. Cardiomyocytes are stained red with phalloidin-Alexa633. Nuclei are stained blue with 4,6-diamidino-2-phenylindole (DAPI).

Next, we studied the relationship between TCs and markers of collagen metabolism. Carboxyterminal telopeptides (ICTP) labelling detects a degradation product of cross-linked collagen type I. The collagen propeptides PINP and PIIINP characterize, respectively, the newly synthesized type I and type III collagens. Representative images showing the relationship between TCs and markers of collagen I metabolism are presented in Figure5. Typical TCs with long Tps were observed in myocardial areas of massive collagen I degradation (Fig.5A and B). In contrast, in areas of newly synthesized collagen I (PINP) only remnants of Tps were observed (Fig.5C and D).

The coefficients of correlations between the number of TCs and Tps with the markers of collagen metabolism are shown in Table2. Correlations between the number of TCs and Tps with the collagen propeptides PINP and PIIINP revealed an inverse relationship. In contrast, the number of TCs and Tps showed a linear increase with the amount of degraded collagen I marker - ICTP. Collectively, these results indicate that a close relationship exists between TCs and the ECM composition.

**Table 2:** Spearman's correlation coefficients of TCs and Tps numbers and collagen metabolism markers in the study groups

	Control (*n* = 6)	DCM (*n* = 6)	ICM (*n* = 7)	MYO (*n* = 6)
TC - ICTP	*r* = 0.779	*r* = 0.825	*r* = 0.789	*r* = 0.826
Tp - ICTP	*r* = 0.899	*r* = 0.898	*r* = 0.827	*r* = 0.861
TC - PINP	*r* = −0.785	*r* = −0.899	*r* = −0.803	*r* = −0.790
Tp - PINP	*r* = −0.868	*r* = −0.923	*r* = −0.818	*r* = −0.861
TC - PIIINP	*r* = −0.728	*r* = −0.831	*r* = −0.811	*r* = −0.922
Tp - PIIINP	*r* = −0.743	*r* = −0.739	*r* = −0.834	*r* = −0.757

ICTP: collagen type I C-terminal telopeptide; PINP: procollagen type I N-terminal propeptide; PIIINP: procollagen type III N-terminal propeptide; ICM: ischemic cardiomyopathy; DCM: dilated cardiomyopathy; MYO: myocarditis.

### The ultrastructure of TCs and Tps in normal and failing human hearts

**Figure 6:**
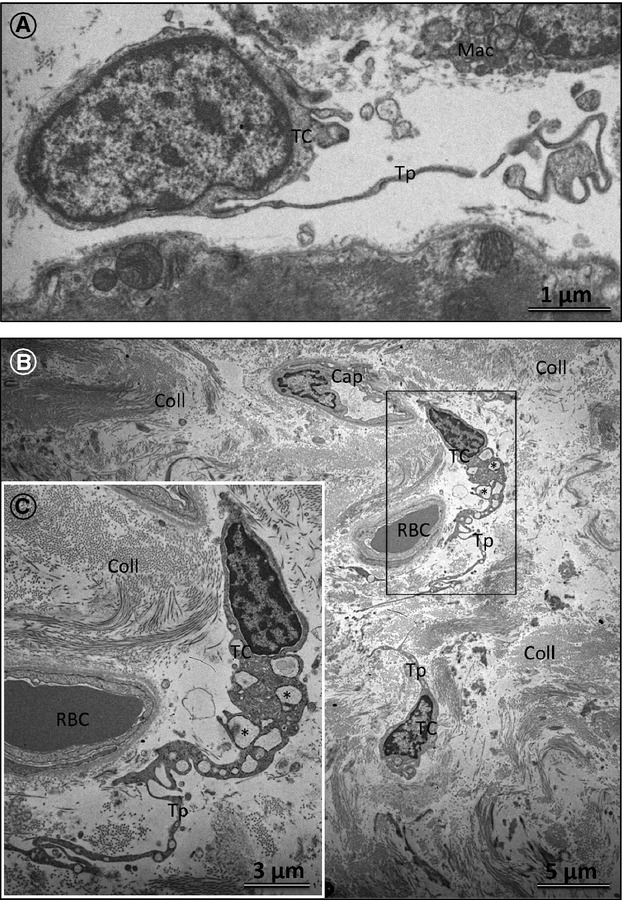
Transmission electron microscopy (TEM) of telocytes (TCs) in normal and diseased human hearts. (**A**) TEM of the normal human LV myocardium showing a typical TC cell body. Note that a telopode (Tp) begins from the cell body abruptly, as a very thin prolongation. Mac: macrophage. (**B**) The ultrastructure of TCs engulfed in a compact area of collagen (Coll) fibrils in a patient with ischemic cardiomyopathy (ICM). Note the marked vacuolization of the TC cytoplasm (asterisk). RBC: red blood cell; Cap: capillary. (**C**) An enlarged view of the boxed region shown in **B**.

The ultrastructure of TCs in normal hearts is very well described. An example of a TC in normal human heart is shown in Figure6A. In diseased human hearts, the TCs are characterized by degenerative processes including cytoplasmic vacuolization, shrinkage and shortening of the Tps (Fig.6B).

**Figure 7:**
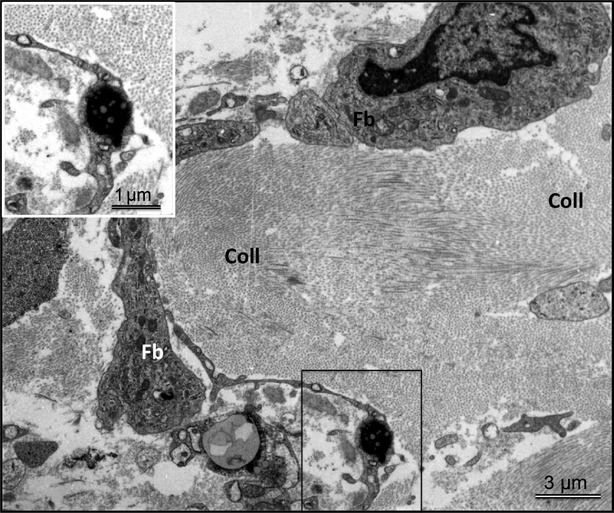
Representative transmission electron microscopy (TEM) of the LV myocardium in a patient with dilated cardiomyopathy showing typical features of a telocyte (TC) undergoing apoptosis (boxed region). Note that apoptotic TC is embedded in densely packed collagen fibres. Mention also that in comparison with a normal TC (Fig.6A), the fibroblast (Fb) shows a large cell body with short and thick processes and is reach in mitochondria and cisternae of rough endoplasmic reticulum.

Most probably, both TC degenerative changes and the ECM composition may lead to TC death. Figure7 shows an apoptotic TC displaying typical ultrastructural features of apoptotic chromatin condensation. Importantly, apoptotic TCs were found to occur mainly in areas of densely packed collagen fibrils. The occurrence of apoptotic TCs found by TEM confirmed the immunohistochemical findings using TUNEL labelling (Fig.3D and E).

**Figure 8:**
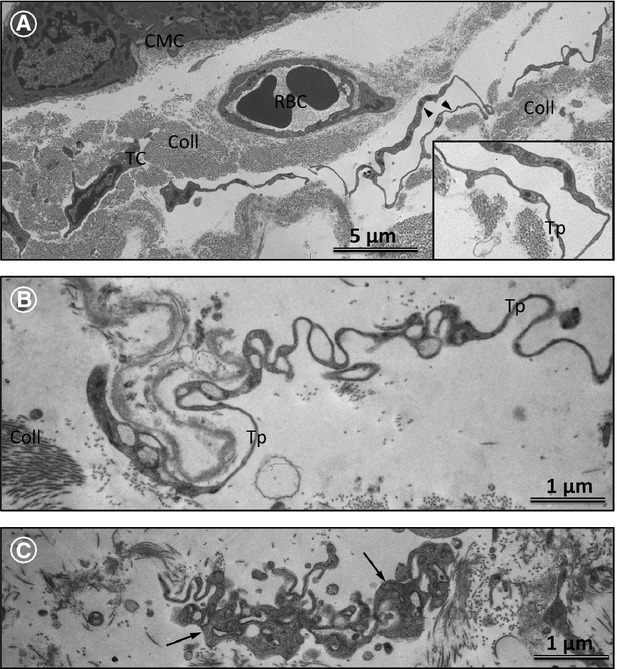
Transmission electron microscopy (TEM) of telocytes (TCs) and telopodes (Tps) in diseased human hearts. (**A**) Representative TEM image showing that in collagen-free areas the TPs show a strong tendency to extend their prolongations and interconnect Tps derived from other TCs (arrowheads). In contrast, the TC engulfed in densely packed collagen fibrils displays retracted Tps. Coll: collagen; CMC: cardiomyocyte; RBC: red blood cell. The insert is a higher magnification of the TP shown with arrowheads in **A**. (**B**) Tps embedded in amorphous, non-fibrillar interstitial material exhibit almost normal ultrastructure and they extend over long distances. (**C**) TEM micrographs showing that in extracellular matrix regions rich in amorphous substances the Tps form typical labyrinthine systems (arrows) usually seen in the normal human myocardium.

The relationship between the ultrastructure of the ECM and TCs is presented in Figure8. In zones of tightly packed collagen fibrils, the TCs show shrinkages of their cytoplasm and Tps (Fig.8A). In contrast, in ECM zones rich in amorphous material, the Tps rebuild their typical normal prolongations (Fig.8A and B). Importantly, in such zones, the TCs and Tps show intact labyrinthine systems (Fig.8C). The TEM results confirm our immunohistochemical data showing that in areas of degraded collagens the number of TCs and Tps is much higher than in areas of replacement fibrosis where mature or newly synthesized fibrillar collagen predominate.

## Discussion

The major finding of this study is that TCs are reduced in the diseased and failing human myocardium. The imbalance between TC proliferation and TC apoptotic death, documented in this study, is responsible for the TCs depletion in cardiac diseases leading to HF. Reduced numbers of TCs have been documented in other cardiac diseases including myocardial infarctions in rats 26, myocardial alterations in systemic sclerosis 24 and in ageing human hearts 35. Significant reductions of TCs have also been shown in liver fibrosis 36, colonic wall in ulcerative colitis 37, the terminal ileum of patients affected by small bowel Crohn's disease 38, gallstone disease 39, endometriosis 40, skin pathologies 24,37,41,42, renal ischemia/reperfusion injury 43 and different lung diseases (reviewed in 44,45).

We have also demonstrated that in diseased human hearts with HF, TCs display severe ultrastructural degenerative changes and undergo apoptotic cell death. The latter was confirmed using both, TEM and TUNEL staining. Similar ultrastructural alterations of TCs and TC apoptosis have been documented in psoriatic dermis 42.

Currently, there are no specific immunohistochemical markers for the identification of cardiac TCs. However, cell morphology and positive expression of c-kit have been used for the identification of cardiac TCs 26,46. In this study, using immunolabelling for c-kit we have found that 1 mm^2^ of the normal human myocardium contains 14.9 ± 3.4 TCs and 41.6 ± 5.9 TPs. The values of c-kit-positive TCs found by Zhao *et al*. 26 in the rat heart ranged from 13.92 ± 2.8 TCs in zones bordering myocardial infarctions to 44.69 ± 1.4 TCs per 1 mm^2^ in the base part of control hearts. In addition, a recent morphometric study of TEM images revealed 22 ± 2 TCs per 1 mm^2^ myocardial area of the neonatal human heart and 19 ± 3 TCs per 1 mm^2^ myocardial area of the adult human heart 35. Taken together, all these data obtained independently by other groups concur well with the number of TCs in human hearts reported in this study and presumably reflect the biologic reality.

The pathophysiological consequences of reduced TCs observed in our patients with different etiologies of HF are largely unknown. It is, however, tempting to speculate that the reduction in TCs in diseased human hearts could participate in the abnormal three-dimensional spatial organization and disturbed intercellular signalling of the myocardium as TCs are considered to be as structurally connecting cells with other heart cells and given that TCs are involved in intercellular signalling *via* homo- or heterocellular cell-to-cell contacts 4,19, shedding microvesicles, exosomes 6,47,48 or paracrine secretion including microRNAs 21. Decreased TCs in HF would be predicted to impair the property of TCs to maintain cardiac stem cell niche 2,21,35 thereby decreasing the pool of cardiac stem cells. This notion is supported by the observations that the number of cardiac stem cell is markedly reduced in diseased human hearts 49. Moreover, the observations that TCs transplantation after myocardial infarction is beneficial for functional regeneration of the infarcted myocardium, demonstrate the therapeutic utility of TCs transplantation in diseased hearts 26.

Another major finding of this study is that the number of TCs is markedly influenced by the composition of the ECM. We have found that in zones of replacement fibrosis or in zones with tightly packed fibrillar collagens, the TCs and Tps are significantly reduced or even absent. Similar observations have been documented in the scar tissue after myocardial infarction 26. In addition, we have shown that in such zones, TCs displayed severe ultrastructural alterations in form of cytoplasmic vacuolization, absence of the TCs labyrinthine components, shrinkage and shortening of the Tps. In contrast, in interstitial areas rich in amorphous material, the TCs are more numerous with the elongation of Tps and formation of multiple TCs labyrinthine components. Our TEM data were confirmed by quantitative confocal microscopy showing that the number of TCs and Tps correlated positively with the degraded collagen type I.

Our findings demonstrating that TCs are influenced by the ECM protein composition are in good agreement with a recent *in vitro* study showing that the dynamics of cardiac Tps are also influenced by the ECM proteins: the stronger Tps spreading being produced by fibronectin, while the lowest by laminin 28. In this study, TCs seeded on collagen determined the highest dynamics of Tps extensions 28. The apparent discrepancy between this and our study stems in the fact that collagen metabolism *in vivo* is a more complex process than that *in vitro*. Despite such a discrepancy, both studies complement each other and add to the conclusion that TCs are influenced by the ECM composition.

## Conclusions

This study demonstrates for the first time that in diseased and failing human hearts the number of TCs is reduced because of a higher rate of apoptosis than in normal hearts. It is also concluded that TCs and Tps in diseased human hearts respond to any quantitative and qualitative changes in the ECM composition such that the number of TCs and Tps correlates negatively with the amount of mature fibrillar collagens and correlates positively with degraded collagens.
